# Global tests of P-values for multifactor dimensionality reduction models in selection of optimal number of target genes

**DOI:** 10.1186/1756-0381-5-3

**Published:** 2012-05-22

**Authors:** Hongying Dai, Madhusudan Bhandary, Mara Becker, J Steven Leeder, Roger Gaedigk, Alison A Motsinger-Reif

**Affiliations:** 1 Department of Medical Research, Children’s Mercy Hospital, 2401 Gillham Road, Kansas City, MO 64108, USA; 2 Department of Mathematics, Columbus State University, 4225 University Avenue, Columbus, GA, 31907, USA; 3 Division of Clinical Pharmacology and Medical Toxicology, Department of Pediatrics, Children’s Mercy Hospital, 2401 Gillham Road, Kansas City, MO 64108, USA; 4 Bioinformatics Research Center, Department of Statistics, North Carolina State University, 1 Lampe Dr, Raleigh, NC, 27695-7566, USA

**Keywords:** P-value, Global tests, ReliefF, Multifactor dimensionality reduction

## Abstract

**Background:**

Multifactor Dimensionality Reduction (MDR) is a popular and successful data mining method developed to characterize and detect nonlinear complex gene-gene interactions (epistasis) that are associated with disease susceptibility. Because MDR uses a combinatorial search strategy to detect interaction, several filtration techniques have been developed to remove genes (SNPs) that have no interactive effects prior to analysis. However, the cutoff values implemented for these filtration methods are arbitrary, therefore different choices of cutoff values will lead to different selections of genes (SNPs).

**Methods:**

We suggest incorporating a global test of p-values to filtration procedures to identify the optimal number of genes/SNPs for further MDR analysis and demonstrate this approach using a ReliefF filter technique. We compare the performance of different global testing procedures in this context, including the Kolmogorov-Smirnov test, the inverse chi-square test, the inverse normal test, the logit test, the Wilcoxon test and Tippett’s test. Additionally we demonstrate the approach on a real data application with a candidate gene study of drug response in Juvenile Idiopathic Arthritis.

**Results:**

Extensive simulation of correlated p-values show that the inverse chi-square test is the most appropriate approach to be incorporated with the screening approach to determine the optimal number of SNPs for the final MDR analysis. The Kolmogorov-Smirnov test has high inflation of Type I errors when p-values are highly correlated or when p-values peak near the center of histogram. Tippett’s test has very low power when the effect size of GxG interactions is small.

**Conclusions:**

The proposed global tests can serve as a screening approach prior to individual tests to prevent false discovery. Strong power in small sample sizes and well controlled Type I error in absence of GxG interactions make global tests highly recommended in epistasis studies.

## Background

Recent advances in genotyping technology have allowed for the rapid and easy interrogation of large numbers of genetic variants for association with common, complex disease. While there have been a number of successes in association mapping studies, the associations found typically explain very little of the overall heritability of the traits being studied. There are several potential reasons for this “missing heritability”, and one of those potential explanations is epistatic interactions (gene-gene interactions). It is hypothesized that such interactions play an important role in the etiology of complex (non-Mendelian) traits, but detecting such interactions presents a number of statistical and computation challenges [1]. In response to these challenges, a number of new data-mining approaches have been developed [2].

Multifactor Dimensionality Reduction (MDR) is a popular and highly successful statistical method developed to detect and characterize nonlinear complex gene-gene or gene-environment interactions (epistasis) that could be associated with disease susceptibility. The method was first proposed by Ritchie et al. [3] to detect estrogen-metabolism gene interactions associated with sporadic breast cancer. MDR has several advantages over more traditional statistical approaches such as logistic regression modeling: 1) MDR is a non-parametric approach with no requirement to the distribution of data. 2) MDR can analyze non-linear associations in genotypic combinations. 3) MDR has improved power to detect gene-gene interaction in small to moderate sample sizes. Since the introduction of the original MDR implementation, many works have been published to improve modeling and prediction accuracy with the MDR method. For more information on the history and development of the method, please refer to the comprehensive review of the MDR and its extended methods by Moore [4].

While the MDR approach is widely used, to make this paper self-contained, we give a brief description of the method. MDR is often applied to genotypic data to detect gene-gene (GxG) interactions among single nucleotide polymorphism (SNP) and the original implementation of this method can be extended to detect the interactions in other types of data when the explanatory variables are categorical variables and the outcome variable is binary. As the scale of association studies has expanded (with larger numbers of SNPs), a filtration step is often implemented in the first step of MDR analysis to remove noisy SNPs. In this step, a subset of genes that are unlikely to interact with others is removed by filtration methods such as SURF [5], TuRF [6] etc. ReliefF [7], has become a commonly applied filter, and we will focus on this filter in the current study. After this step, the remaining SNPs are used for the dimensionality reduction and model selection steps of the MDR algorithm. In this step, all variable combinations are considered for k-way (*k* = 2, 3, 4 …) interactions. For each multi-locus combination, the ratio of cases to controls within each contingency table cell is calculated, and then each cell is assigned a status of high-risk or low-risk by comparing this ratio to the ratio of cases: controls in the overall dataset. Cells with a ratio greater than the overall ratio are assigned “high-risk” status, and those with a ratio lower than the overall ratio are assigned “low-risk” status. Subsequently, a balanced classification accuracy is calculated for each multi-locus combination, and the optimal model is selected based on the highest balanced accuracy. This model selection approach is performed in concert with a cross-validation procedure, usually 10-fold, which randomly divides the whole data set into a training set and a validation set. The testing accuracy is the balanced accuracy when the classification rule developed from the training data set is applied to the testing data set. The cross validation count (CVC) summarizes the number of times a model is the top model in each of the cross-validation splits of the data. The optimal *k*-way (*k* = 2, 3, 4 …) interaction model with the highest training accuracy and the highest CVC is then selected as the winner model. Finally, the significance of the selected optimal model is assessed by permutation testing (comparing the testing/prediction accuracy against the empirical distribution built by at least 1000 permutations). MDR can be performed by an open source software mdr2.0 and model goodness-of-fit and significance can be assessed using software mdrpt1.0 [8] or in the MDR.R R software package [9].

In this work, we seek to address two existing issues in the current MDR analysis. First, current filtration approaches do not evaluate the significance of the SNPs considered (or provide p-value for their measures) and there is no clear guideline for the cutoff point of such filtration measures. This leads inconsistency in the optimal number of SNPs remaining for the final MDR analysis.

Second, as there is a growing appreciation that the etiology of human diseases is extremely complex, many investigators are using MDR to evaluate many potential interactive effects, and not just a single final best model [10]. In this type of approach, not one but numerous tests can be performed in search of an optimal model in the *k*-way interaction, as the number of partitions for *k*-way interaction over *m* loci is $\left(\begin{array}{l} m\\ k \end{array}\right)=\frac{m!}{k!(m-k)!}$. For instance, if an investigator is interested to detect significant 2-way interactions among 50 SNPs, 1225 tests will be performed which will inflate the family-wise Type I error rate to $1-{\left(1-\alpha \right)}^{\left(\begin{array}{l} m\\ k \end{array}\right)}=1-{\left(1-0.05\right)}^{1225}\approx 1$, where α=0.05 is the nominal error rate for an individual test without proper control.

False discoveries and losing power to detect the signal after the multiplicity adjustment are two concurrent issues in analyzing high dimensional data. Instead of replacing all the existing methods to control the false discovery, we propose to add the global tests to the current MDR framework as an ad-hoc screening process to prevent false discoveries. We will explain the rationale and utility of global tests in Section Global tests.

Incorporating global tests within the filtration procedures can reveal a trend of gene interactive patterns when noisy genes (SNPs) are removed step by step using ReliefF or other filtration techniques. In the current study, we demonstrate this approach in a candidate gene study of drug response in Juvenile Idiopathic Arthritis, using the ReleifF filter. Additionally, we perform a simulation study comparing several different global testing approaches for a range of genetic etiologies to compare the power of different approaches.

## Methods

### Global tests

**Figure 1:**
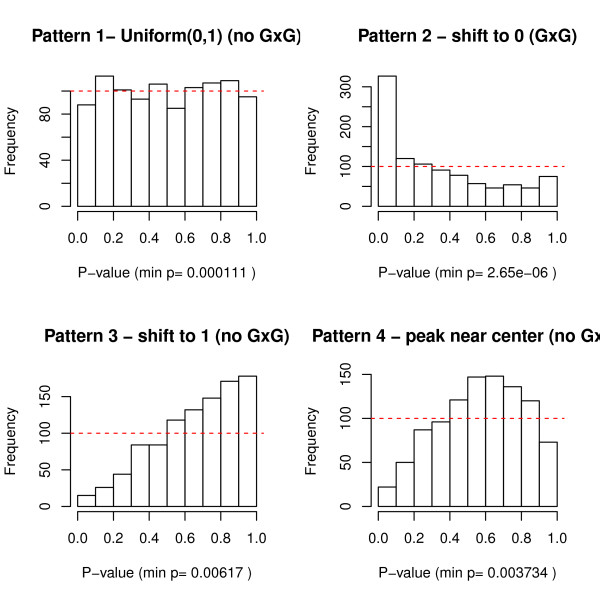
Four patterns of p-values.

The idea of global testing is to assess the patterns of p-values from multiple testing of *k* way interactions among *m* loci ($n=\left(\begin{array}{l} m\\ k \end{array}\right)=\frac{m!}{k!(m-k)!}$tests). Under the null hypothesis of no GxG interactions, the p-values will follow uniform (0, 1). To see this, let *T* be the test statistic with the cumulative distribution function (CDF) *F*_0_*(t)* and the inverse CDF $F_{0}^{-1}(t)$ for t∈Runder *H*_*0*_. Let *P* be the p-value corresponding to the test statistic *T*. Under *H*_*0*_, we have $\mathrm{Pr}(P\leq p)=\mathrm{Pr}(F_{0}^{-1}(P)\leq F_{0}^{-1}(p))=F_{0}(F_{0}^{-1}(p))=p$ for p∈(0,1) (Pattern 1 in Figure 1).

When a proportion of *m* loci have *k-*way interactions (*H*_*α*_), it is expected to observe the p-values shifting towards 0. To see this, let *F(t)* be the CDF under *H*_*α*_ and $F(t)>F_{0}(t)$ for t∈R, then $\mathrm{Pr}(P\leq p)=\mathrm{Pr}(F_{0}^{-1}(P))\leq F_{0}^{-1}(p))=F(F_{0}^{-1}(p))>p$ for p∈(0,1) (Pattern 2 in Figure 1). Due to correlations/linkage disequilibrium among SNPs and the redundancy of SNPs in high order models, sometimes p-values shift toward 1, i.e. Pr(P≤p)<p for p∈(0,1)(Pattern 3 in Figure 1). When p-values are correlated, they might peak near center of histogram (Pattern 4 in Figure 1). Patterns 3 and 4 are deviated from uniformity but they do not indicate potential *k-*way interactions among *m* loci.

The rationale of global testing is to ensure p-values are not randomly and uniformly distributed (Pattern 1) before we investigate each single p-value. Correlated p-values without significant effects (*H*_0_) might even shift toward 1 or peak near the center (Patterns 3 and 4). The goals are to rule out Patterns 1, 3 and 4 and only move forward to the final MDR analysis when p-values are in Pattern 2.

If the entire set of p-value follows a uniform distribution, then it is very likely for a small p-value to be a false discovery by chance. As shown in Figure 1, the entire set of p-values might have four different Patterns: uniform, shifting to 0, shifting to 1 or peak near the center. In all four cases, we notice that the minimum p-values are less than 0.05 (0.0001111 in Pattern 1, 2.65e-6 in Pattern 2, 0.00617 in Pattern 3 and 0.003734 in Pattern 4). If we take the distribution of the entire set of p-values into account, then the minimum p-values in Patterns 1, 3 and 4 are false discoveries by chance.

### Combined global testing and filtration technique

A global test will serve as an ad-hoc diagnostic tool to exam all p-values from *k-*way interactions among *m* genes in MDR-analysis. These p-values come from empirical distributions generated through permutation testing. Let $P_{}=p_{i}(i=1\text{,}\;2\text{,}\cdots \text{,}\;n)$be identical and independently distributed (i.i.d.) p-values from the MDR analysis of *k–way* interactions among *m–*loci ($n=\left(\begin{array}{l} m\\ k \end{array}\right)=\frac{m!}{k!(m-k)!}$). We will consider a one-sided test to compare

(17) $$\left\{ \begin{array}{l} H_{0}:P\overset{}{\sim }Uniform(0,1)\\ versus\\ H_{a}:\mathrm{Pr}(P\leq p)>p\;for\;p\in (0,1) \end{array}\right.$$

Rejecting *H*_0_ indicates significant GxG interactions in some target genes.

We propose incorporating global testing of p-values with ReliefF [7] gene filtration technique to detect the patterns of *k*-way GxG interaction among *m* genes (SNPs) and remove noisy genes (SNPs) with little interactive effects to determine the optimal number of SNPs for the final MDR analysis. The ReliefF algorithm estimates weights to measure the potential accuracy of attributes in prediction of phenotype. The redundant attribute will be assigned a lower score. When applied in gene-gene interactions, a higher ReliefF score indicates a stronger interactive effect for the corresponding gene (SNP). ReliefF algorithm first uses *x*- nearest neighborhood approach (x=1,2,⋯,m) to match a selected subject with *x* subjects in neighborhood (with shortest distances across all SNPs) from the control group and from test group respectively. An attribute (SNP) will be assigned score 1 (−1) if the attribute from the selected subject matches (mismatches) one of *x* nearest subjects from the same phenotype group. Similarly, an attribute will be assigned score −1 (1) if the attribute from the selected subject matches (mismatches) one of the nearest subjects from the different phenotype group. The score will be aggregated for all subjects and normalized (divided) by the total number of subjects and neighbors. Detailed description of ReliefF algorithm for filtering genotyping data can be found in Section 3 of [4].

**Figure 2:**
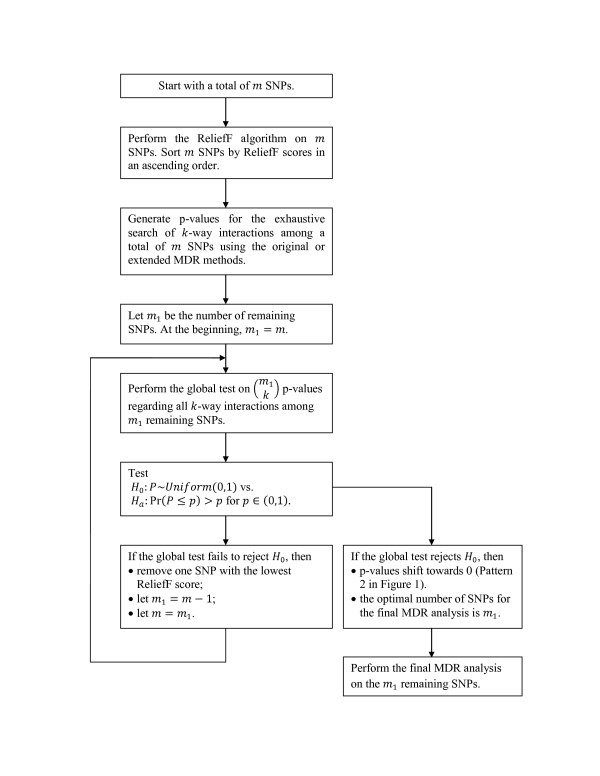
Flow chart of global testing of p-values in conjunction with filtration process.

The flow chart of the testing procedure is presented in Figure 2. Starting with a set of *m* candidate SNPs, perform the ReliefF algorithm on *m* SNPs and sort SNPs by ReliefF scores in an ascending order. Generate p-values for the exhaustive search of *k-*way interactions among a total of *m* SNPs using the original or extended MDR methods. For each *k-*way interaction, one p-value of MDR analysis is generated by permutation test. Let *m*_*1*_ be the number of remaining SNPs. Perform the global testing on $\left(\begin{array}{l} m_{1}\\ k \end{array}\right)$ p-values. Remove one SNP that has the lowest ReliefF score and all interactions corresponding to this SNP. Perform the global testing about hypothesis testing (1) on the p-values of the *k*-way interactions of the remaining SNPs. Continue to remove SNPs with the lowest ReliefF score one by one and perform global testing after each removal of a SNP. One can stop the process when the remaining SNPs reach a predetermined minimum number. Choose the optimal number of SNPs for the final MDR analysis as the largest number of SNPs with global testing p-value *< α*. Often we set α=0.05. To be more rigorous of controlling family-wise Type I error, one can apply FDR algorithm on the global testing p-values and the first p-value with FDR < 0.05 will determine the optimal number of SNPs.

### Global tests of P-values

Here we introduce 6 global tests that can be applied to test hypothesis (1). These six tests are based on different approaches to detect deviation from uniformity. We will survey these methods and compare their power using a case study and Monte-Carlo simulations.

### Test 1 one sided Kolmogorov-Smirnov test [KS]

KS test is a non-parametric test that can be applied to compare the distance between an empirical distribution of i.i.d. p-values and Uniform(0, 1). For hypothesis test (1), define the one-sided KS statistic as $D_{n}^{+}=\underset{p}{\sup}\left(\frac{1}{n}\sum_{i=1}^{n}I_{\left\{p_{i}\leq p\right\}}-p\right)$, where $I_{\left\{p_{i}\leq p\right\}}$is an indicator function which equals 1 if $p_{i}\leq p$and 0 if $p_{i}>p$. According to [11], the p-value of one-sided KS test follows $\mathrm{Pr}\left(D_{n}^{+}>t\right)=t\sum_{i=0}^{\left[n(1-t)\right]}\left(\begin{array}{l} n\\ i \end{array}\right){\left(1-t-i/n\right)}^{n-i}{\left(t+i/n\right)}^{i-1}$ where [n(1−t)] is the largest integer not greater than n(1−t) and t∈(0,1).

### Test 2 one-sided inverse chi-square test [inverse chi]

Fisher [12] shows that if $p_{i}\overset{i.i.d.}{\sim }Uniform(0,1)$ for i=1,2,⋯,n, then $-2\sum_{i=1}^{n}\ln(p_{i})\sim \chi_{2n}^{2}$where $\chi_{2n}^{2}$is chi-square distribution with 2*n* degrees of freedom. For a one sided test (1), reject *H*_0_ if $-2\sum_{i=1}^{n}\ln(p_{i})>\chi_{2n,1-\alpha }^{2}$where $\chi_{2n,1-\alpha }^{2}$ is (1−α)*100%percentile of $\chi_{2n}^{2}$.

### Test 3 one sided inverse normal test [inverse norm]

Transform p-value to normal z score by letting $z_{i}=\Phi^{-1}(p_{i})$where $\Phi^{-1}$ is inverse cumulative normal distribution. Under *H*_*0*_, $Z=\left(\sum_{i=1}^{n}z_{i}\right)/\sqrt{n}\sim N(0\text{,}\;1)$. For one sided test (1), reject *H*_0_ if *Z < Z*_*α*_ where *Z*_*α*_ is α*100% percentile of the standard normal distribution.

### Test 4 one sided logit test [logit]

Logit transform p-value by letting $L=\sum_{i=1}^{n}\ln(p_{i}/(1-p_{i}))$. [13] shows that under *H*_0_, the distribution of *L* can be closely approximated by Student’s t-distribution with 5n+4degrees of freedom, namely $L^{*}=L\sqrt{\frac{3(5n+4)}{\pi^{2}n(5n+2)}}\approx t_{5n+4}$. Therefore, for one-sided test (1), we can reject *H*_0_ if $L^{*}<t_{5n+4,\alpha }$where $t_{5n+4,\alpha }$ is α*100% percentile of the t-distribution.

### Test 5 one sided Wilcoxon test [Wilcoxon]

Order *n* p-values from MDR testing along with *n*_*2*_ observations randomly drawn from Uniform(0,1) from least to greatest and denote them by $S_{1}\text{,}\;S_{2}\text{,}\cdots \text{,}\;S_{N}$ with $N=n+n_{2}$. Let *W* be the sum of the ranks corresponding to *n* p-values from MDR testing. For one-sided test (1), we can reject *H*_0_ if $W\leq n(N+1)-\omega_{\alpha }$where the constant *ω*_*α*_ is chosen to make the Type I error probability equal *α*. Values of *ω*_*α*_ are given in Table A6 by [14]. For large sample sizes, i.e. min(*n,n*_2_) going to infinity, one can apply normal approximation on the standardized *W*.

### Test 6 Tippett and Wilkinson’s test [Tippett]

Tippett’s Test [15] is based on the property of the minimal p-value in multiple testing. Let $p_{\left(1\right)}\text{,}\;p_{\left(2\right)}\text{,}\cdots \text{,}\;p_{\left(n\right)}$be the ordered p-values in an ascending order. When p-values identically and independently follow *Uniform*(0,1) distribution, Tippett’s test will reject *H*_0_ if $p_{\left(1\right)}<1-{\left(1-\alpha \right)}^{1/n}$. The p-value of Tippett’s test equals $1-{\left(1-p_{\left(1\right)}\right)}^{n}$. Tippett’s test is very easy to perform but it only takes the smallest p-value into account.

Wilkinson [16] extended Tippett’s procedure to the *r*^*th*^ smallest p-values where r=1,2,⋯,n. By expanding ${\left(\alpha +(1-\alpha )\right)}^{n}$, Wilkinson tabulated the probability, denoted by *C*_*γ,α*_ of obtaining *r* significant statistics by chance in a group of *n* tests. Suppose there are *r* tests with p-values less than *α*, Wilkinson’s test rejects *H*_0_ if $c_{r,\alpha }<\alpha$[17]. Because *P*_*(r)*_ has been distributed with parameters r and n-r + 1, tables of the incomplete beta function can be used to obtain critical values of *P*_*(r)*_ directly. In our work, we will not include Wilkinson’s test in case study and power simulation because this method does not provide p-value for the testing results.

### Case study

We used a real dataset to illustrate how to apply our proposed global testing to prevent false discovery and to determine the optimal number of SNPs for the final MDR analysis. Juvenile Idiopathic Arthritis (JIA) is one of the most common chronic diseases of childhood, affecting an estimated 300,000 children in the U.S. alone, and is an important cause of morbidity and disability in children [18]. Although methotrexate (MTX) is the most commonly used second-line agent used to treat JIA worldwide, this antifolate drug has shown considerable inter-individual variability in clinical response and adverse reactions [19]. The polyglutamation of methotrexate (MTXglu) is an intracellular mechanism that retains the drug and enhances target enzyme inhibition within the folate pathway [20], and high concentrations of “long chain” methotrexate polyglutamates (MTXglu_3-5_) have been associated with improved response to the drug in adults with rheumatoid arthritis [21]. Studies have reported the extensive variability in intracellular MTXglu concentrations in JIA, and an association of long chain MTXglu with toxicity (but not efficacy) in children [22]. Due to the complexity of the folate cycle as well as the extensive variability in response to the drug in clinical practice, it is hypothesized that genetic factors may contribute to differences seen in distinct patterns of MTXglu concentrations intracellularly, which might further impact patients’ responses to MTX.

In this case study, we analyzed 25 SNPs from 17 candidate genes in the folate pathway (Table 1). MTXglu was measured in all patients after at least 3 months on stable MTX therapy and a range of 1 to 5 glutamate moieties were reported as a percentage of the total polyglutamate concentration (MTXglu_n%_). Hierarchical clustering was performed to identify patterns of MTXglu_n%_^,^ and two clusters were determined based on the hierarchical clustering of normalized MTXglu_1-5%_. Subjects in cluster 1 had lower concentration of short chain polyglutamates (MTXglu_1-2%_) and higher concentration of long chain polyglutamates (MTXglu_3-5%_) as compared to subjects in cluster 2 (p < 0.05). These clusters reflected distinct patterns in the proportion of MTXglu concentrations.

**Table 1:** List of 25 SNPs from 17 candidate genes in the folate pathway

**SNP**	**RS #**	MAF*
*ABCG2 C > T*	rs7699188	0.13
*ABCG2 15846 A > C*	no rs [35]	0.01
*ABCG2 G > A*	rs35252139	0.13
*ABCG2 A > G*	rs35229708	0.13
*ABCG2 C > T*	55930652	0.27
*ATIC C > T*	rs12995526	0.3
*BHMT A > G*	rs3733890	0.33
*DHFR A > T*	rs7387	0.3
*GGH C > T*	rs3758149	0.27
*MTHFD2 indel*	rs71391718	0.31
*MTHFR C > T*	rs1801133	0.3
*MTHFR A > C*	rs1801131	0.33
*MTHFR G > A*	rs2274976	0.06
*MTR A > G*	rs1805087	0.19
*MTRR A > G*	rs1801394	0.57
*SHMT1 C > T*	rs1979277	0.37
TYMS *2/*3	rs34743033	0.49
*TYMS indel*	rs11280056	0.32
*FOLH1 C > T*	rs61886492	0.03
*GART A > G*	rs8788	0.21
*GART A > G*	rs8971	0.19
*SLC25A32 G > A*	rs17803441	0.07
*ADORA2a C > T*	rs2298383	0.61
*ITPA T > C*	rs2295553	0.52
*SLCO1B1 T > C*	rs4149056	0.12

*Minor Allele Frequency.

There were 30 subjects in Cluster 1 and 74 subjects in Cluster 2. The MTXglu clustering phenotype was coded 1 and 0 for MDR analysis. Genotypes, coded 0 for common homozygote, 1 for heterozygote and 2 for rare homozygote for 25 SNPs, were measured. The overall goal of the analysis was to assess whether interactions among SNPs are associated with MTXglu clustering. While the scale of this study is not so large that an exhaustive search of all SNPs is computationally limited, this data is used to demonstrate the proposed approach.

For illustrative purposes, we will focus on 2-way interactions among 25 SNPs and will determine the optimal number of targeted SNPs for testing 2-way interactions. We first applied the ReliefF algorithm to 25 SNPs. As shown in Table 2, ReliefF scores ranged from −0.0308 to 0.1163. Although a higher score indicates stronger interaction with other SNPs, there is no clear cutoff point for ReliefF scores.

**Table 2:** FDR adjusted P-value in global testing

**# SNPs**	**Remaining**	**SNP**	**ReliefF**	**P-values of global testing**					
Removed	SNP (GxG)	Removed	Score	KS	Inverse chi	Inversenorm	Logit	Wilcoxon	Tippett
0	25(300)			0.29121	0.22008	1.00000	1.00000	0.80991	0.33927
1	24(276)	rs35252139	−0.0308	0.21644	0.12424	1.00000	1.00000	0.55397	0.33340
2	23(253)	rs35229708	−0.0308	0.16697	0.06603	1.00000	1.00000	0.43198	0.32718
3	22(231)	rs2298383	−0.0279	0.13417	0.03087	1.00000	1.00000	0.25182	0.32062
4	21(210)	rs12995526	−0.0250	0.16987	0.05667	1.00000	1.00000	0.45717	0.31374
5	20(190)	rs7699188	−0.0202	0.11033	0.01793	1.00000	1.00000	0.31680	0.30658
6	19(171)	rs1805087	−0.0183	0.03887	0.00320	1.00000	1.00000	0.18665	0.29916
7	18(153)	rs4149056	−0.0067	0.02048	0.00106	1.00000	1.00000	0.09019	0.29154
8	17(136)	55930652	−0.0038	0.01051	0.00080	1.00000	1.00000	0.10254	0.28376
9	16(120)	* no rs [35]	−0.0029	0.00455	0.00016	1.00000	1.00000	0.02642	0.27592
10	15(105)	rs17803441	0.0010	0.00312	0.00011	1.00000	1.00000	0.00567	0.26811
11	14(91)	rs34743033	0.0087	0.00022	0.00001	1.00000	1.00000	0.00015	0.26049
12	13(78)	rs61886492	0.0096	0.00022	0.00000	0.00001	0.00000	0.00006	0.25328
13	12(66)	rs71391718	0.0183	0.00008	0.00000	0.00000	0.00000	0.00001	0.25328
14	11(55)	rs3758149	0.0192	0.00008	0.00000	0.00000	0.00000	0.00006	0.25328
15	10(45)	rs1801131	0.0240	0.00026	0.00004	0.00004	0.00004	0.00016	0.25328
16	9(36)	rs1801394	0.0365	0.00022	0.00004	0.00003	0.00003	0.00015	0.25328
17	8(28)	rs8788	0.0375	0.00008	0.00003	0.00002	0.00002	0.00015	0.25328
18	7(21)	rs7387	0.0452	0.01051	0.00255	0.00547	0.00442	0.00799	0.25328
19	6(15)	rs1801133	0.0481	0.05212	0.06679	0.10791	0.12185	0.05337	0.25328
20	5(10)	rs8971	0.0481						
21	4(6)	rs2274976	0.0644						
22	3(3)	rs1979277	0.0673						
23	2(1)	rs11280056	0.0702						
24		rs3733890	0.0750						
25		rs3733890	0.1163						
**Optimal number of SNPs removed**				5	4	11	11	8	Not Found

To circumvent this limitation, we incorporated global testing of p-values and ReliefF algorithm using the method proposed in Section Combined global testing and filtration technique. We first generated p-values for all 2-way interactions among 25 SNPs through permutation testing. Then we applied global testing, including KS test, Inverse chi test, Inverse norm test, Logit test, Wilcoxon test and Tippett’s tests on $\left(\begin{array}{l} 25\\ 2 \end{array}\right)=300$ p-values of 2-way interactions among 25 SNPs. The global tests were performed to evaluate whether the distribution of p-values deviated from uniformity (null hypothesis) and shifted towards 0 (alternative hypothesis - Pattern 2 in Figure 1). Then we removed one SNP with the lowest ReliefF score step by step and repeated the global testing process until only 5 SNPs were remained. We stopped the global testing procedure at 5 SNPs because it is not meaningful or necessary to perform global testing when the number of SNPs is less than 5 in any case study.

The entire procedure of filtration and global testing of p-values are summarized in Table 2. The optimal number of SNPs is the largest number of SNPs with global testing p-value <0.05. In this case study, KS test and Inverse chi test were more sensitive to deviation from uniformity as the tests became significant after 5 and 4 SNPs removed respectively (Table 2). Inverse norm test and Logit test were more conservative, suggesting removal of 11 SNPs. Wilcoxon test, removing 8 SNPs, was moderate as compared to the other tests. Tippett’s test failed to detect significant GxG interactions with all FDR corrected p-values > 0.05. Our further simulation studies (discussed in Section Power simulation) indicate that Tippett’s test, which only takes the smallest p-value into account, might not be appropriate for global testing of p-values.

**Figure 3:**
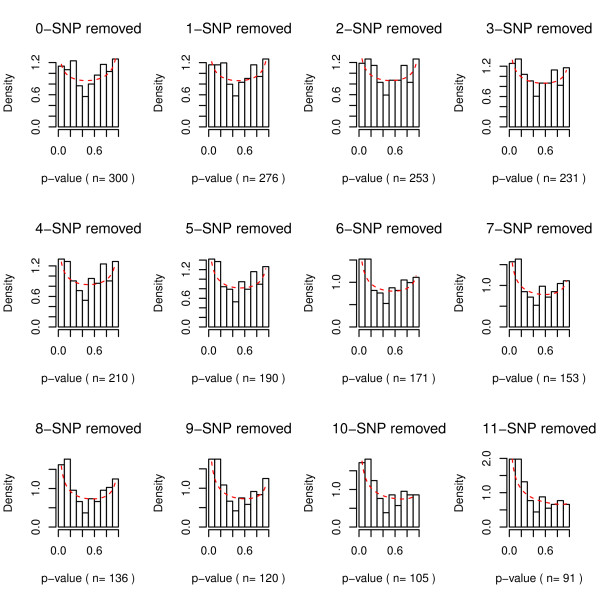
**Histograms of p-values from multiple tests GxG interactions (Genes with weak interactive effects/low ReliefF scores are removed step by step.** The red dash curves are fitted beta density functions).

**Figure 4:**
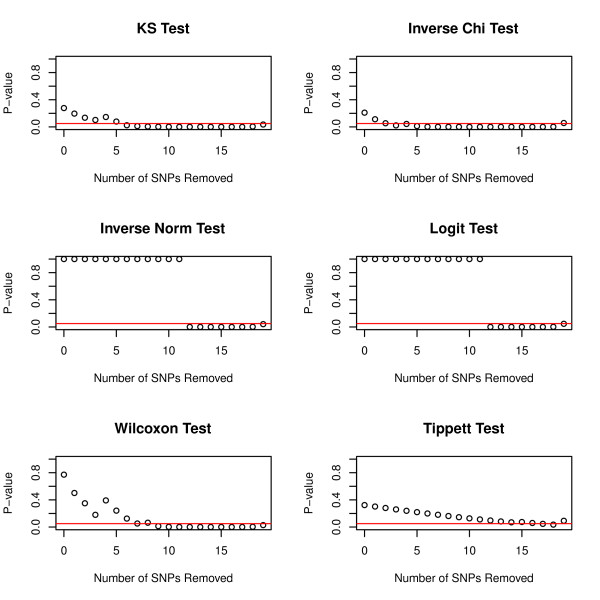
**Global Testing of p-values combined with filtration technique (The red line is at nominal rate 0.05.** The optimal number of genes is determined when the global test first has p-value < 0.05).

Figure 3 and 4 both revealed strong patterns of transition when noisy SNPs were removed. In Figure 3, as SNPs with low ReliefF scores were removed sequentially, the histogram of p-values started to shift toward 0. Also in Figure 4, the global test p-values were in decreasing trends when noisy SNPs were removed one by one. Once the p-value of global testing was under 0.05, it continued to stay under 0.05. There were only a few exceptions at the end of the filtration procedure, probably due to smaller sample sizes and reduction of power.

**Figure 5:**
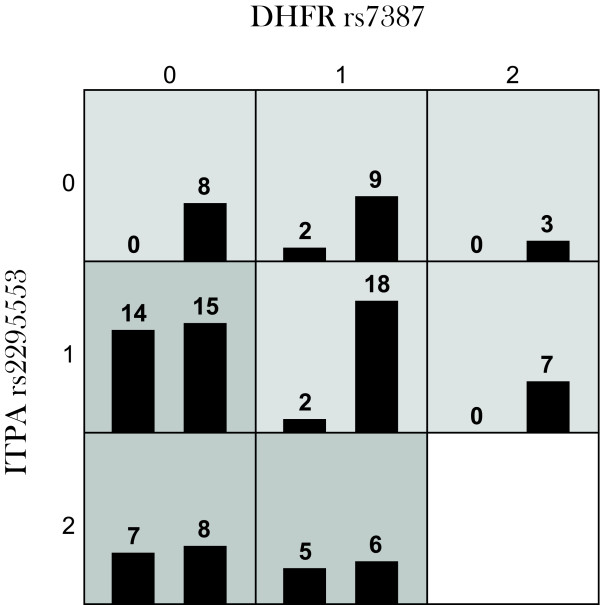
**Gene to gene interaction detected by MDR after filtering out 6 SNPs according to the one sided inverse chi-square test.** (The distribution of MTXglu clustering among genotypic combinations between DHFR-rs7387 and ITPA-rs2295553 is listed. The genotype for DHFR-rs7387 and ITPA-rs2295553 is coded as 0-homozygote, 1-heterozygote and 2-rare homozygote. In each cell, the first column stands for the number of subjects in cluster 1 (low concentration of MTXglu_1-2_% and high concentration of MTXglu_3-4_%) and the second column stands for the number of subjects in cluster 2(high concentration of MTXglu_1-2_% and low concentration of MTXglu_3-5_%_._ Genotypic combinations in relatively high (low) likelihood of cluster 1 are displayed in darkly (lightly) shaded cells).

After the filtration and global testing, we removed 4 SNPs as suggested by Inverse chi test with FDR correction (Table 2) and performed MDR analysis on the remaining 21 SNPs. The results of MDR analysis indicated that there was significant two-way interaction between *DHFR* (rs7387) and *ITPA* (rs2295553) with testing balance accuracy = 0.7374 (p = 0.0045). The MDR analysis was performed by an open source software mdr2.0 and model goodness-of-fit and significance was assessed by permutation using software mdrpt1.0 [8].

The dihydrofolate reductase (*DHFR*) enzyme is a well known important target of MTX action. When *DHFR* is inhibited by MTX the subsequent production of reduced folates such as tetrahydrofolate (*THF*) 5,10 methenyl-THF and 5-methyl-THF are altered, affecting not only total cellular folate concentrations but also the downstream effects from one carbon donation including homocysteine remethylation and pyrimidine and purine synthesis thought to result in an anti-proliferative effect [23]. The inhibition of *DHFR* also results in a buildup of the precursor dihydrofolate (DHF) which in its polyglutamated state has inhibitory effects upon enzymes within the pathway as well [24]. Inosine triphosphate pyrophosphatase (*ITPA*) plays a role in *de novo* purine synthesis, and is closely related to adenosine metabolism, which is thought to contribute to MTX response via its anti-inflammatory effect [25]. Variations in *ITPA* interestingly have been shown by other authors to contribute to MTX response as part of a candidate gene study in JIA [26], as well as “predisposing genetic attribute” in studies utilizing MDR in adults with rheumatoid arthritis [27,28]. How these 2 genes directly affect MTXglu patterns remains difficult to determine, as the direct understanding of how MTXglu patterns are associated with response is yet to be elucidated. However, both genes encode enzymes closely linked to or directly affected by MTX, thus as we gain a more detailed knowledge of cellular folate metabolism and its disruption by anti-folate agents such as MTX, we will then develop a better understanding of this complex system, and how alterations in the folate pathway affect response to the drug.

### Power simulation

In the two empirical studies described below, we investigate the performance of 6 global testing when p-values exhibit different patterns (Figure 1) of variation. Data were generated from the Uniform distribution or varying mixtures of Beta distributions based on the inference regarding the patterns of p-values as described in Section Global tests and Figure 1. Here we give the rationale of using uniform or beta mixture to simulate p-values under null and alternative hypotheses. Under the null hypothesis of no GxG interactions, we have proved that p-values follow Uniform(0,1) distribution (Pattern 1). When this null hypothesis is violated, we introduce a latent variable to indicate the status of underlying hypothesis for each test. For $p_{i}\text{,}\;i=1\text{,}\;2\text{,}\cdots \text{,}\;n$, introduce a latent variable $Z_{i}$ where for hypothesis testing (1), we have

(2) $$\left\{ \begin{array}{l} Z_{i}=0\;\text{if}\;H_{0}\text{:}\mathrm{no}\;\text{GxG}\;\text{for}\;\text{the}\;i^{\mathrm{th}}\;\text{test}(\text{Pattern}\;1\text{,}\;\mathrm{Pr}(P\leq p)=p)\\ Z_{i}=1\;if\;H_{a}:\text{GxG}\;\text{for}\;\text{the}\;i^{\mathrm{th}}\;\text{test}(\text{Pattern}\;2\text{,}\;\mathrm{Pr}(P\leq p)>p)\text{,}\; \end{array}\right.$$

for p∈(0,1). The proportion of tests where *H*_*α*_ holds is denoted by the mixing weight $\text{Pr}(Z_{i}=1)=\pi$ where π∈(0,1).

Conditioning on *Z*_*i*_, we have

(3) $$\left\{ \begin{array}{l} p_{i}|Z_{i}=0\sim \text{Uniform}(0,1)\\ p_{i}|Z_{i}=1\sim \text{Beta}(a,\;b)\text{where}\;a>0\;\text{and}\;b>0\text{,}(a\text{,}\;b)\neq (1\text{,}\;1)\text{.} \end{array}\right.$$

The marginal distribution of combined p-values becomes P~(1−π)Uniform(0,1)+πBeta(a,b), which indicates that with (1−π)×100% of chance, a p-value is drawn from *Uniform*(0,1) and with π×100% of chance, a p-value is drawn from *Beta(α,b)*. Beta distribution is very flexible to characterize the patterns of p-values (Figure 1) where Uniform (0,1) is a special case of Beta (1,1). One can also adjust the shape and scale parameters *a* and *b* to model the deviation from uniformity.

The p-values from MDR analysis are correlated due to linkage disequilibrium among SNPs and sharing the SNPs among GxG interactions. The dependence among p-values might cause inflation of Type I errors or lead to bias in global tests. As a result, it is critical to extensively simulate p-values with varying correlation structures and assess the robustness of global tests for correlated p-values. In this work, we simulated correlated Uniform variables with random correlation matrix Σ and Beta random variables with correlation coefficient ρ=0.2,0.8,Beta(2,5),Uniform(0.1,0.9) respectively. The details of generating correlated uniform [29] and beta distributions [30] are summarized in Appendix 1.

The first simulation study concerns the Type I error of global testing when there does not exist any GxG interactions among genes (SNPs). We generated p-values from

· Independent Uniform(0,1),

· Correlated Uniform(0,1),

· Correlated 0.9Uniform(0,1)+0.1Beta(5,1), (4.1)

· Correlated 0.5Uniform(0,1)+0.5Beta(5,1), (4.2)

· Correlated 0.9Uniform(0,1)+0.1Beta(6,3), (4.3)

· Correlated 0.5Uniform(0,1)+0.5Beta(6,3). (4.4)

These six scenarios cover Patterns 1, 3 and 4 with no signs of GxG interactions in Figure 1. For each simulation, the sample size of p-values varies from 20 to 500 and we performed global tests on each sample of p-values. We repeated the process 1000 times, and calculate the percentage of rejection of null hypothesis for each test. Under the hypothesis of no GxG interaction, this rejection rate is considered as Type I error. As shown in Table 3, the Type I error rates are well controlled to be near or under the nominal rate 0.05 when p-values are i.i.d Uniform(0,1). When p-values are correlated Uniform with random correlation matrices, there was slight inflation in five global tests except Tippett’s test. It is good to notice that the inflation is not severe as most tests have Type I error rates under 0.07. Such mild inflation is acceptable in screening testing and we will discuss how to further address this issue in Discussion Section.

**Table 3:** Type I error of six global tests of p-values when p-values are independent or strongly correlated (The nominal Type I error rate is 0.05 and the severe inflation of Type I error with simulated error rate > 0.1 is written in bold italic)

**Independent Uniform (0,1)**						
**n**	**KS**	**Inverse chi**	**Inverse norm**	**Tippett**	**Wilcoxon**	**Logit**
20	0.052	0.053	0.049	0.051	0.052	0.050
50	0.051	0.052	0.051	0.048	0.051	0.050
100	0.046	0.049	0.050	0.049	0.049	0.051
200	0.047	0.048	0.047	0.052	0.049	0.048
300	0.051	0.054	0.053	0.051	0.053	0.053
400	0.042	0.046	0.046	0.048	0.047	0.046
500	0.051	0.050	0.049	0.051	0.050	0.050
**Correlated Uniform (0,1**)						
**n**	**KS**	**Inverse chi**	**Inverse norm**	**Tippett**	**Wilcoxon**	**Logit**
20	0.061	0.059	0.063	0.050	0.065	0.062
50	0.058	0.060	0.063	0.049	0.061	0.062
100	0.060	0.066	0.068	0.049	0.066	0.067
200	0.063	0.069	0.073	0.050	0.072	0.072
300	0.069	0.071	0.074	0.052	0.073	0.074
400	0.064	0.066	0.073	0.048	0.071	0.072
500	0.064	0.070	0.071	0.049	0.068	0.069
**Correlated Uniform (0,1) ± 0.1Beta (5,1),**ρ=0.8						
**n**	**KS**	**Inverse chi**	**Inverse norm**	**Tippett**	**Wilcoxon**	**Logit**
20	0.061	0.047	0.058	0.035	0.061	0.057
50	0.081	0.037	0.054	0.046	0.06	0.053
100	0.080	0.039	0.061	0.045	0.056	0.057
200	***0.156***	0.031	0.062	0.039	0.059	0.059
300	***0.180***	0.017	0.04	0.039	0.049	0.042
400	***0.270***	0.025	0.055	0.052	0.053	0.056
500	***0.278***	0.017	0.052	0.046	0.060	0.052
**Correlated 0.5 Uniform (0,1) ± 0.5Beta (5,1),**ρ=0.8						
**n**	**KS**	**Inverse chi**	**Inverse norm**	**Tippett**	**Wilcoxon**	**Logit**
20	***0.239***	0.007	0.009	0.035	0.013	0.009
50	***0.652***	0.002	0.022	0.025	0.02	0.022
100	***0.911***	0.001	0.018	0.023	0.018	0.019
200	***0.998***	0	0.021	0.034	0.025	0.021
300	***1***	0	0.029	0.033	0.039	0.027
400	***1***	0	0.027	0.024	0.034	0.023
500	***1***	0	0.031	0.024	0.036	0.025
**Correlated 0.9 Uniform (0,1) ± 0.1Beta (6,3),**ρ=0.8						
**n**	**KS**	**Inverse chi**	**Inverse norm**	**Tippett**	**Wilcoxon**	**Logit**
20	0.072	0.041	0.05	0.038	0.052	0.05
50	***0.107***	0.039	0.065	0.051	0.068	0.062
100	***0.148***	0.048	0.086	0.04	0.089	0.086
200	***0.227***	0.038	0.088	0.048	0.101	0.086
300	***0.296***	0.024	0.097	0.042	***0.119***	0.095
400	***0.353***	0.04	***0.108***	0.053	***0.131***	***0.105***
500	***0.439***	0.041	***0.12***	0.033	***0.148***	***0.111***
**Correlated 0.5 Uniform (0,1) ± 0.5Beta (6,3),**ρ=0.8						
**n**	**KS**	**Inverse chi**	**Inverse norm**	**Tippett**	**Wilcoxon**	**Logit**
20	***0.421***	0.01	0.038	0.025	0.063	0.029
50	***0.837***	0.008	0.089	0.019	***0.147***	0.071
100	***0.989***	0.008	***0.16***	0.024	***0.253***	***0.122***
200	***1***	0.001	***0.268***	0.023	***0.411***	***0.207***
300	***1***	0	***0.392***	0.022	***0.581***	***0.32***
400	***1***	0.004	***0.492***	0.031	***0.679***	***0.422***
500	***1***	0.003	***0.551***	0.024	***0.761***	***0.486***

In addition to Uniform distributions, we also simulated correlated Beta mixtures in formula (4.1)-(4.4) regarding p-values shifting to 1 or peaking near the center (Patterns 3 and 4). We conservatively set correlation coefficient ρ=0.8 (Table 3) to simulate very strong correlation among Beta variates, which is most likely to inflate Type I errors in global tests. The simulation results for mild correlation including ρ=0.2,Beta(2,5),Uniform(0.1,0.9)are summarized in Appendix 1 (Table 4). The results from Table 3 and Appendix 1 (Table 4) show that Inverse chi test and Tippett’s test are very robust to dependency in p-values with well controlled Type I error rates. The KS test has the highest inflation in several scenarios we simulated, especially when correlated p-values had peaks near center (Pattern 4). For strongly correlated p-values (ρ=0.8 Table 3), the inverse norm, the Wilcoxon and Logit tests also had modest inflations when sample sizes get larger (n > 200). When p-values were moderately correlated (ρ=0.2,Beta(2,5),Uniform(0.1,0.9), (Table 4)), the inverse norm, the Wilcoxon and the Logit tests had well controlled Type I errors for all tested sample sizes.

**Table 4:** Type I error of six global tests of p-values when p-values are moderately correlated (The nominal Type I error rate is 0.05 and the severe inflation of Type I error with simulated error rate > 0.1 is written in bold italic)

**Correlated Uniform (0,1) ± 0.1Beta (5,1), **ρ=0.2						
**n**	**KS**	**Inverse chi**	**Inverse norm**	**Tippett**	**Wilcoxon**	**Logit**
20	0.018	0.031	0.022	0.048	0.021	0.026
50	0.014	0.019	0.008	0.046	0.01	0.013
100	0.014	0.014	0.013	0.05	0.014	0.014
200	0.003	0.01	0.006	0.044	0.004	0.007
300	0.002	0.005	0.002	0.05	0.001	0.002
400	0.001	0.003	0	0.045	0	0
500	0.002	0.002	0.003	0.042	0.001	0.003
**Correlated 0.5 Uniform (0,1) ± 0.5Beta (5,1),**ρ=0.2						
**n**	**KS**	**Inverse chi**	**Inverse norm**	**Tippett**	**Wilcoxon**	**Logit**
20	0	0	0	0.023	0	0
50	0	0	0	0.023	0	0
100	0	0	0	0.029	0	0
200	0	0	0	0.025	0	0
300	0	0	0	0.024	0	0
400	0	0	0	0.021	0	0
500	0	0	0	0.024	0	0
**Correlated 0.9 Uniform (0,1) ± 0.1Beta (6,3),**ρ=0.2						
**n**	**KS**	**Inverse chi**	**Inverse norm**	**Tippett**	**Wilcoxon**	**Logit**
20	0.041	0.051	0.047	0.047	0.042	0.049
50	0.032	0.049	0.043	0.042	0.034	0.048
100	0.033	0.032	0.035	0.043	0.035	0.036
200	0.017	0.009	0.023	0.052	0.017	0.024
300	0.017	0.016	0.015	0.039	0.011	0.016
400	0.028	0.006	0.014	0.044	0.01	0.017
500	0.021	0.007	0.013	0.048	0.009	0.015
**Correlated 0.5 Uniform (0,1) ± 0.5Beta (6,3),**ρ=0.2						
**n**	**KS**	**Inverse chi**	**Inverse norm**	**Tippett**	**Wilcoxon**	**Logit**
20	0.008	0.004	0.003	0.025	0.001	0.003
50	0.021	0.002	0.001	0.024	0.001	0.002
100	0.046	0.001	0.001	0.028	0	0.001
200	***0.165***	0	0	0.023	0	0
300	***0.337***	0	0	0.025	0	0
400	***0.558***	0	0	0.017	0	0
500	***0.66***	0	0	0.018	0	0
**Correlated Uniform (0,1) ± 0.1Beta (5,1),**ρ=Beta(2,5)						
**n**	**KS**	**Inverse chi**	**Inverse norm**	**Tippett**	**Wilcoxon**	**Logit**
20	0.034	0.043	0.036	0.047	0.034	0.04
50	0.019	0.022	0.02	0.05	0.018	0.021
100	0.012	0.018	0.011	0.038	0.008	0.012
200	0.002	0.008	0.006	0.045	0.006	0.006
300	0.005	0.009	0.005	0.037	0.004	0.007
400	0.004	0.005	0.002	0.038	0.002	0.002
500	0	0.003	0.001	0.05	0	0.002
**Correlated 0.5 Uniform (0,1) ± 0.5Beta (5,1),**ρ=Beta(2,5)						
**n**	**KS**	**Inverse chi**	**Inverse norm**	**Tippett**	**Wilcoxon**	**Logit**
20	0.001	0.002	0.001	0.021	0	0.001
50	0	0	0	0.026	0	0
100	0	0	0	0.016	0	0
200	0	0	0	0.02	0	0
300	0	0	0	0.031	0	0
400	0.002	0	0	0.02	0	0
500	0.001	0	0	0.028	0	0
**Correlated 0.9 Uniform (0,1) ± 0.1Beta (6,3),**ρ=Beta(2,5)						
**n**	**KS**	**Inverse chi**	**Inverse norm**	**Tippett**	**Wilcoxon**	**Logit**
20	0.049	0.046	0.054	0.044	0.055	0.05
50	0.039	0.031	0.042	0.042	0.036	0.043
100	0.027	0.027	0.036	0.05	0.033	0.037
200	0.033	0.023	0.03	0.041	0.025	0.032
300	0.042	0.018	0.029	0.038	0.023	0.032
400	0.041	0.012	0.02	0.046	0.017	0.021
500	0.053	0.013	0.026	0.054	0.02	0.028
**Correlated 0.5 Uniform (0,1) ± 0.5Beta (6,3)**, ρ=Beta(2,5)						
**n**	**KS**	**Inverse chi**	**Inverse norm**	**Tippett**	**Wilcoxon**	**Logit**
20	0.02	0.006	0.008	0.03	0.006	0.009
50	0.051	0	0	0.031	0	0
100	***0.123***	0	0.003	0.023	0.001	0.004
200	***0.271***	0	0	0.021	0	0
300	***0.414***	0	0	0.033	0	0
400	***0.552***	0	0	0.024	0	0
500	***0.663***	0	0	0.028	0	0
**Correlated Uniform (0,1) ± 0.1Beta (5,1),**ρ=Uniform(0.1,0.9)						
**n**	**KS**	**Inverse chi**	**Inverse norm**	**Tippett**	**Wilcoxon**	**Logit**
20	0.036	0.041	0.034	0.048	0.031	0.038
50	0.042	0.039	0.043	0.054	0.039	0.048
100	0.028	0.03	0.029	0.054	0.028	0.029
200	0.037	0.018	0.023	0.045	0.021	0.025
300	0.016	0.012	0.013	0.025	0.015	0.014
400	0.034	0.008	0.017	0.045	0.015	0.019
500	0.031	0.006	0.014	0.046	0.005	0.016
**Correlated 0.5 Uniform (0,1) ± 0.5Beta (5,1),**ρ=Uniform(0.1,0.9)						
**n**	**KS**	**Inverse chi**	**Inverse norm**	**Tippett**	**Wilcoxon**	**Logit**
20	0.011	0.005	0.002	0.017	0.001	0.002
50	0.029	0.002	0.003	0.022	0.001	0.003
100	0.048	0	0	0.023	0.001	0.001
200	0.099	0	0	0.023	0.000	0
300	***0.161***	0	0	0.026	0.000	0
400	***0.176***	0	0	0.016	0.000	0
500	***0.218***	0	0	0.024	0.001	0
**Correlated 0.9 Uniform (0,1) ± 0.1Beta (6,3),**ρ=Uniform(0.1,0.9)						
**n**	**KS**	**Inverse chi**	**Inverse norm**	**Tippett**	**Wilcoxon**	**Logit**
20	0.05	0.036	0.038	0.033	0.044	0.039
50	0.054	0.031	0.047	0.043	0.045	0.044
100	0.044	0.031	0.048	0.05	0.045	0.056
200	0.091	0.034	0.057	0.047	0.058	0.058
300	***0.117***	0.024	0.054	0.049	0.054	0.05 s8
400	0.097	0.022	0.048	0.048	0.048	0.05
500	***0.116***	0.014	0.042	0.035	0.043	0.043
**Correlated 0.5 Uniform (0,1) ± 0.5Beta (5,1),**ρ=Uniform(0.1,0.9)						
**n**	**KS**	**Inverse chi**	**Inverse norm**	**Tippett**	**Wilcoxon**	**Logit**
20	***0.108***	0.009	0.016	0.027	0.017	0.016
50	***0.298***	0.002	0.014	0.025	0.013	0.014
100	***0.459***	0.002	0.014	0.024	0.016	0.014
200	***0.774***	0	0.016	0.017	0.022	0.017
300	***0.908***	0	0.016	0.023	0.023	0.015
400	***0.933***	0	0.024	0.024	0.029	0.019
500	***0.975***	0	0.016	0.02	0.032	0.013

Uniform distributions have random correlation matrices. The nominal Type I error rate is 0.05 and the severe inflation of Type I error with simulated error rate > 0.1 is written in bold italic**).**

In the second simulation study, we are interested in the power of each of the approaches to detect the GxG interactions by performing the hypothesis testing (1) to detect Pr(P≤p)>p(Pattern 2 with GxG interactions). We simulated p-values from a wide range of beta mixture distribution where the mixing *π* was set to be 0.1 and 0.4, indicating different proportions of tests with significant GxG interactions. In most cases, parameters a<bwill have Pr(P≤p)>pfor p∈(0,1) which coincides with Pattern 2. Under alternative hypothesis of a proportion of tests having GxG interaction, we simulated p-values from 6 Beta mixtures:

· Correlated 0.9Uniform(0,1)+0.1Beta(0.4,6),

· Correlated 0.6Uniform(0,1)+0.4Beta(0.4,6),

· Correlated 0.9Uniform(0,1)+0.1Beta(0.5,4.5),

· Correlated 0.6Uniform(0,1)+0.4Beta(0.5,4.5),

· Correlated 0.9Uniform(0,1)+0.1Beta(1,5), and

· Correlated 0.6Uniform(0,1)+0.4Beta(1,5).

As in the Type I error simulation, in the power simulation, we used sample sizes ranging from 20 to 500 and perform simulation 1000 times. The power is the percentage of results across the 1000 replicates where the null hypothesis was rejected. We present the power comparison results under week correlation ρ=0.2 in Table 5 because all six global tests are free of Type I errors in this scenario. Power comparisons for ρ=0.8,Beta(2,5),Uniform(0.1,0.9) are summarized in Appendix 1 (Table 6). These results indicate that Tippett’s test, which only takes the smallest p-value into account, might not be appropriate for detecting the patterns of alternation. Among the other five global tests, Inverse chi test has the strongest power in most simulated cases. These five global tests have strong power to detect Pr(P≤p)>pfor small to moderate sample sizes.

**Table 5:** **Power of six global tests of correlated P-values (The correlation coefficient for Beta random variables is***ρ***. Uniform distributions have random correlation matrices)**

**Correlated 0.9Uniform (0,1) ± 0.1Beta (0.4,6), **ρ=0.2						
**n**	**KS**	**Inverse chi**	**Inverse norm**	**Tippett**	**Wilcoxon**	**Logit**
20	0.176	0.359	0.259	0.332	0.191	0.294
50	0.27	0.576	0.418	0.505	0.303	0.464
100	0.407	0.824	0.599	0.652	0.456	0.672
200	0.645	0.967	0.827	0.791	0.657	0.877
300	0.808	0.995	0.92	0.861	0.785	0.941
400	0.896	0.997	0.966	0.896	0.861	0.979
500	0.949	0.999	0.984	0.933	0.926	0.993
**Correlated 0.6 Uniform (0,1) ± 0.4Beta (0.4,6),**ρ=0.2						
**n**	**KS**	**Inverse chi**	**Inverse norm**	**Tippett**	**Wilcoxon**	**Logit**
20	0.797	0.957	0.896	0.784	0.815	0.918
50	0.987	1	0.997	0.939	0.983	1
100	1	1	1	0.972	1	1
200	1	1	1	0.998	1	1
300	1	1	1	1	1	1
400	1	1	1	0.999	1	1
500	1	1	1	0.999	1	1
**Correlated 0.9 Uniform (0,1) ± 0.1Beta (0.5,4.5),**ρ=0.2						
**n**	**KS**	**Inverse chi**	**Inverse norm**	**Tippett**	**Wilcoxon**	**Logit**
20	0.151	0.266	0.207	0.225	0.183	0.221
50	0.227	0.466	0.336	0.326	0.264	0.369
100	0.343	0.664	0.493	0.437	0.397	0.534
200	0.545	0.863	0.709	0.537	0.595	0.757
300	0.706	0.944	0.832	0.587	0.719	0.863
400	0.811	0.982	0.913	0.632	0.81	0.933
500	0.89	0.992	0.951	0.695	0.887	0.966
**Correlated 0.6 Uniform (0,1) ± 0.4Beta (0.5,4.5),**ρ=0.2						
**n**	**KS**	**Inverse chi**	**Inverse norm**	**Tippett**	**Wilcoxon**	**Logit**
20	0.161	0.291	0.212	0.225	0.173	0.237
50	0.251	0.475	0.362	0.302	0.292	0.385
100	0.322	0.64	0.504	0.397	0.39	0.528
200	0.517	0.882	0.701	0.527	0.58	0.743
300	0.726	0.952	0.845	0.568	0.731	0.873
400	0.806	0.976	0.894	0.607	0.805	0.917
500	0.895	0.998	0.95	0.676	0.883	0.965
**Correlated 0.9 Uniform (0,1) ± 0.1Beta (1,5),**ρ=0.2						
**n**	**KS**	**Inverse chi**	**Inverse norm**	**Tippett**	**Wilcoxon**	**Logit**
20	0.127	0.118	0.139	0.056	0.144	0.136
50	0.203	0.199	0.211	0.063	0.235	0.206
100	0.248	0.272	0.28	0.064	0.271	0.269
200	0.406	0.448	0.453	0.06	0.446	0.438
300	0.535	0.559	0.541	0.073	0.542	0.535
400	0.619	0.657	0.649	0.07	0.657	0.631
500	0.725	0.743	0.729	0.071	0.731	0.715
**Correlated 0.6 Uniform (0,1) ± 0.4Beta (1,5),**ρ=0.2						
**n**	**KS**	**Inverse chi**	**Inverse norm**	**Tippett**	**Wilcoxon**	**Logit**
20	0.572	0.56	0.599	0.117	0.611	0.587
50	0.893	0.864	0.904	0.104	0.917	0.891
100	0.991	0.981	0.986	0.114	0.991	0.981
200	1	1	0.999	0.091	1	0.999
300	1	1	1	0.109	1	1
400	1	1	1	0.111	1	1
500	1	1	1	0.085	1	1

**Table 6:** **Power of six global tests of correlated P-values (The correlation coefficient for Beta random variables is** ***ρ*** **Uniform distributions have random correlation matrices)**

**Correlated 0.9 Uniform (0,1)** **±** **0.1Beta (0.4,6),**ρ=0.8						
**n**	**KS**	**Inverse chi**	**Inverse norm**	**Tippett**	**Wilcoxon**	**Logit**
20	0.19	0.402	0.285	0.329	0.204	0.323
50	0.293	0.654	0.458	0.547	0.324	0.514
100	0.41	0.833	0.644	0.665	0.48	0.7
200	0.705	0.963	0.856	0.785	0.693	0.901
300	0.864	0.989	0.943	0.861	0.834	0.956
400	0.934	0.996	0.967	0.878	0.862	0.978
500	0.962	0.999	0.989	0.914	0.928	0.994
**Correlated 0.6 Uniform (0,1)** **±** **0.4Beta (0.4,6),**ρ=0.8						
**n**	**KS**	**Inverse chi**	**Inverse norm**	**Tippett**	**Wilcoxon**	**Logit**
20	0.848	0.968	0.913	0.787	0.837	0.924
50	0.994	0.999	0.998	0.922	0.989	0.998
100	1	1	0.999	0.956	0.999	0.999
200	1	1	1	0.987	1	1
300	1	1	1	0.993	1	1
400	1	1	1	0.992	1	1
500	1	1	1	0.994	1	1
**Correlated 0.9 Uniform (0,1)** **±** **0.1Beta (0.5,4.5),**ρ=0.8						
**n**	**KS**	**Inverse chi**	**Inverse norm**	**Tippett**	**Wilcoxon**	**Logit**
20	0.145	0.282	0.199	0.243	0.158	0.237
50	0.281	0.491	0.376	0.345	0.296	0.409
100	0.374	0.686	0.532	0.425	0.418	0.573
200	0.598	0.874	0.734	0.531	0.627	0.771
300	0.774	0.951	0.866	0.579	0.767	0.89
400	0.855	0.975	0.914	0.641	0.836	0.936
500	0.938	0.993	0.962	0.635	0.91	0.977
**Correlated 0.6 Uniform (0,1)** **±** **0.4Beta (0.5,4.5),**ρ=0.8						
**n**	**KS**	**Inverse chi**	**Inverse norm**	**Tippett**	**Wilcoxon**	**Logit**
20	0.149	0.287	0.221	0.217	0.178	0.242
50	0.265	0.518	0.4	0.334	0.323	0.438
100	0.421	0.693	0.539	0.434	0.438	0.575
200	0.604	0.856	0.726	0.485	0.644	0.754
300	0.778	0.951	0.858	0.582	0.761	0.886
400	0.871	0.978	0.931	0.612	0.858	0.942
500	0.921	0.981	0.948	0.658	0.901	0.957
**Correlated 0.9 Uniform (0,1)** **±** **0.1Beta (1,5),**ρ=0.8						
**n**	**KS**	**Inverse chi**	**Inverse norm**	**Tippett**	**Wilcoxon**	**Logit**
20	0.157	0.147	0.158	0.066	0.154	0.154
50	0.192	0.234	0.231	0.069	0.222	0.22
100	0.32	0.355	0.347	0.064	0.344	0.34
200	0.491	0.521	0.501	0.061	0.502	0.488
300	0.655	0.698	0.663	0.068	0.651	0.652
400	0.776	0.784	0.754	0.068	0.751	0.741
500	0.847	0.844	0.82	0.072	0.823	0.802
**Correlated 0.6 Uniform(0,1)** **±** **0.4Beta(1,5),**ρ=0.8						
**n**	**KS**	**Inverse chi**	**Inverse norm**	**Tippett**	**Wilcoxon**	**Logit**
20	0.698	0.676	0.695	0.124	0.703	0.682
50	0.966	0.921	0.938	0.131	0.948	0.922
100	0.999	0.988	0.991	0.11	0.997	0.988
200	1	1	1	0.116	1	1
300	1	1	1	0.122	1	1
400	1	1	1	0.122	1	1
500	1	1	1	0.114	1	1
**Correlated 0.9 Uniform(0,1)** **±** **0.1Beta(0.4,6),**ρ=Beta(2,5)						
**n**	**KS**	**Inverse chi**	**Inverse norm**	**Tippett**	**Wilcoxon**	**Logit**
20	0.147	0.349	0.245	0.332	0.181	0.288
50	0.263	0.59	0.422	0.529	0.298	0.481
100	0.396	0.804	0.627	0.648	0.447	0.687
200	0.675	0.963	0.831	0.804	0.662	0.89
300	0.809	0.99	0.927	0.836	0.808	0.951
400	0.899	0.998	0.966	0.907	0.865	0.979
500	0.958	0.999	0.98	0.928	0.912	0.99
**Correlated 0.6 Uniform(0,1)** **±** **0.4Beta(0.4,6),**ρ=Beta(2,5)						
**n**	**KS**	**Inverse chi**	**Inverse norm**	**Tippett**	**Wilcoxon**	**Logit**
20	0.827	0.974	0.921	0.789	0.808	0.941
50	0.994	1	0.999	0.945	0.977	0.999
100	1	1	1	0.983	1	1
200	1	1	1	0.997	1	1
300	1	1	1	1	1	1
400	1	1	1	1	1	1
500	1	1	1	0.999	1	1
**Correlated 0.9 Uniform(0,1)** **±** **0.1Beta(0.5,4.5),**ρ=Beta(2,5)						
**n**	**KS**	**Inverse chi**	**Inverse norm**	**Tippett**	**Wilcoxon**	**Logit**
20	0.141	0.269	0.205	0.229	0.162	0.231
50	0.235	0.444	0.33	0.321	0.257	0.367
100	0.359	0.661	0.526	0.408	0.423	0.562
200	0.543	0.879	0.709	0.516	0.577	0.747
300	0.726	0.95	0.849	0.562	0.731	0.878
400	0.81	0.977	0.902	0.632	0.81	0.928
500	0.914	0.993	0.959	0.683	0.895	0.967
**Correlated 0.6 Uniform (0,1)** **±** **0.4Beta (0.5,4.5),**ρ=Beta(2,5)						
**n**	**KS**	**Inverse chi**	**Inverse norm**	**Tippett**	**Wilcoxon**	**Logit**
20	0.138	0.26	0.199	0.238	0.169	0.212
50	0.241	0.469	0.356	0.335	0.288	0.385
100	0.347	0.662	0.506	0.402	0.408	0.55
200	0.547	0.885	0.703	0.519	0.585	0.761
300	0.694	0.949	0.852	0.592	0.727	0.88
400	0.802	0.987	0.914	0.627	0.804	0.935
500	0.882	0.991	0.948	0.641	0.871	0.959
**Correlated 0.9 Uniform(0,1)** **±** **0.1Beta(1,5),**ρ=Beta(2,5)						
**n**	**KS**	**Inverse chi**	**Inverse norm**	**Tippett**	**Wilcoxon**	**Logit**
20	0.121	0.118	0.133	0.071	0.132	0.129
50	0.194	0.21	0.214	0.064	0.217	0.211
100	0.277	0.311	0.305	0.071	0.306	0.299
200	0.449	0.502	0.486	0.068	0.485	0.477
300	0.585	0.643	0.612	0.058	0.623	0.599
400	0.66	0.72	0.697	0.075	0.696	0.682
500	0.767	0.8	0.775	0.059	0.759	0.759
**Correlated 0.6 Uniform(0,1)** **±** **0.4Beta(1,5),**ρ=Beta(2,5)						
**n**	**KS**	**Inverse chi**	**Inverse norm**	**Tippett**	**Wilcoxon**	**Logit**
20	0.594	0.591	0.632	0.124	0.637	0.616
50	0.913	0.9	0.921	0.139	0.922	0.914
100	0.996	0.992	0.992	0.111	0.995	0.99
200	1	1	1	0.102	1	1
300	1	1	1	0.115	1	1
400	1	1	1	0.116	1	1
500	1	1	1	0.095	1	1
**Correlated 0.9 Uniform(0,1)** **±** **0.1Beta(0.4,6),**ρ=Uniform(0.1,0.9)						
**n**	**KS**	**Inverse chi**	**Inverse norm**	**Tippett**	**Wilcoxon**	**Logit**
20	0.183	0.397	0.292	0.363	0.217	0.324
50	0.291	0.639	0.459	0.545	0.34	0.508
100	0.418	0.821	0.625	0.653	0.463	0.694
200	0.669	0.958	0.855	0.78	0.68	0.881
300	0.818	0.993	0.924	0.861	0.782	0.947
400	0.919	0.997	0.97	0.908	0.89	0.985
500	0.974	1	0.988	0.926	0.932	0.994
**Correlated 0.6 Uniform(0,1)** **±** **0.4Beta(0.4,6),**ρ=Uniform(0.1,0.9)						
**n**	**KS**	**Inverse chi**	**Inverse norm**	**Tippett**	**Wilcoxon**	**Logit**
20	0.815	0.964	0.928	0.825	0.836	0.945
50	0.99	1	0.997	0.92	0.988	0.997
100	1	1	1	0.971	1	1
200	1	1	1	0.993	1	1
300	1	1	1	0.997	1	1
400	1	1	1	0.998	1	1
500	1	1	1	1	1	1
**Correlated 0.9 Uniform(0,1)** **±** **0.1Beta(0.5,4.5),**ρ=Uniform(0.1,0.9)						
**n**	**KS**	**Inverse chi**	**Inverse norm**	**Tippett**	**Wilcoxon**	**Logit**
20	0.154	0.283	0.216	0.228	0.171	0.24
50	0.272	0.465	0.352	0.331	0.296	0.391
100	0.358	0.675	0.512	0.421	0.414	0.561
200	0.528	0.871	0.706	0.526	0.578	0.749
300	0.749	0.958	0.857	0.578	0.752	0.89
400	0.857	0.986	0.924	0.632	0.841	0.938
500	0.906	0.99	0.951	0.679	0.877	0.962
**Correlated 0.6 Uniform(0,1)** **±** **0.4Beta(0.5,4.5),**ρ=Uniform(0.1,0.9)						
**n**	**KS**	**Inverse chi**	**Inverse norm**	**Tippett**	**Wilcoxon**	**Logit**
20	0.137	0.282	0.2	0.228	0.159	0.234
50	0.237	0.484	0.359	0.306	0.293	0.392
100	0.345	0.679	0.503	0.411	0.416	0.544
200	0.577	0.858	0.719	0.512	0.6	0.757
300	0.725	0.946	0.848	0.584	0.743	0.873
400	0.868	0.981	0.92	0.621	0.85	0.931
500	0.9	0.989	0.947	0.676	0.869	0.959
**Correlated 0.9 Uniform(0,1)** **±** **0.1Beta(1,5),**ρ=Uniform(0.1,0.9)						
**n**	**KS**	**Inverse chi**	**Inverse norm**	**Tippett**	**Wilcoxon**	**Logit**
20	0.141	0.166	0.16	0.078	0.155	0.155
50	0.213	0.224	0.235	0.054	0.251	0.232
100	0.318	0.362	0.364	0.073	0.365	0.362
200	0.477	0.525	0.498	0.047	0.503	0.485
300	0.621	0.651	0.637	0.071	0.622	0.621
400	0.718	0.747	0.721	0.071	0.708	0.707
500	0.804	0.818	0.794	0.075	0.785	0.78
**Correlated 0.6 Uniform(0,1)** **±** **0.4Beta(1,5),**ρ=Uniform(0.1,0.9)						
**n**	**KS**	**Inverse chi**	**Inverse norm**	**Tippett**	**Wilcoxon**	**Logit**
20	0.694	0.656	0.705	0.109	0.721	0.685
50	0.938	0.901	0.929	0.125	0.94	0.915
100	0.994	0.989	0.991	0.114	0.995	0.99
200	1	1	1	0.143	1	1
300	1	0.999	1	0.113	1	1
400	1	1	1	0.122	1	1
500	1	1	1	0.119	1	1

Uniform distributions have random correlation matrices).

The global tests have been implemented in R. The R code is available at http://www.childrensmercy.org/Content/view.aspx?id=22812.

## Discussion and conclusions

Multifactor Dimensionality Reduction (MDR) is a novel statistical method developed to characterize and detect nonlinear complex gene-gene interactions (epistasis) that could be associated with disease susceptibility. We suggest incorporating global test to filtration procedures to reveal a trend of gene interactive patterns when noisy genes are removed step by step using ReliefF or other filtration techniques. The optimal number of genes for further MDR analysis can be identified by p-values of global testing. A real data applications and empirical assessment of our proposed methods reveal strong trends in global testing of p-values and clear patterns of distribution of p-values in three scenarios: 1) presence of GxG interactions, 2) absence of GxG interactions, 3) weak GxG interactions that needs filtration to remove noisy genes. The proposed global tests can serve as a screening approach before individual tests to prevent false discovery. Strong power in small sample sizes and well controlled Type I error in absence of GxG interactions makes these tests highly recommended in epistasis studies.

Global testing has not been implemented in MDR analyses in the literature we have reviewed. Currently, researchers rely on adjustment of individual p-values such as false discovery rate (FDR) as suggested by [31]. Due to high dimensionality in genetic interactions, the FDR and other multiple testing adjustments often lose power in MDR analyses. Some MDR studies [27] have utilized the false positive report probability proposed by [32] but this method has been pointed out by [33] to be heuristic and wrong in formulation. In contrast, the global tests proposed by this paper are based on rigorous statistical theories and inferences.

Through extensive simulation on correlated p-values, our study shows that the Inverse chi test is the most powerful approach to be incorporated with the filtration techniques to determine the optimal number of SNPs for the final MDR analysis. The KS test might have high inflation of Type I errors when p-values are highly correlated or when p-values peak near the center of histogram (Pattern 4). The Tippett’s test has very low power when the effect size of Pattern 2 is small.

We observe mild inflation of Type I error (<0.07) when p-values are Uniform with a random correlation matrix. Our global tests are implemented for screening SNPs and investigators can continue to use multiplicity adjustment algorithms such as FDR to adjust individual p-values in the final MDR analysis to prevent false discoveries. As a result, slight inflation in Type I error (<0.07) is acceptable in practice. Moreover, in our case study, we show that one can utilize the decreasing trend of global test results (Figure 4) to facilitate decision making. If global tests provided false discoveries, then the trend of global tests results would randomly fluctuate up and down. Figure 4 with a decreasing trend for global testing results as well Figure 3 with histograms systematically switching to Pattern 2 can also serve as diagnostic tools to prevent false discoveries or selection bias in global tests.

It is worthwhile to point out the proposed global tests can effectively prevent false discovery without losing the power to detect significant GxG interactions. To prevent the false discovery, current MDR applications typically choose one optimal model for each k-way interaction. This method has two major drawbacks: firstly, the false positive discovery is not reduced by choosing one optimal model; secondly, choosing one optimal models may overlook other potential GxG interactions that also contributes to the disease susceptibility.

The major contribution of our manuscript is to incorporate global testing procedures to MDR framework. Our proposed global tests will provide p-values to help practitioners determine the appropriate number of SNPs to be remained in the final analysis. The current filtration process does not provide p-values. Therefore, using arbitrary cutoff value in the current process might lead to over-filtering or under-filtering of SNPs.

All 6 global tests are based on statistical inference instead of permutation. These 6 tests run very fast in a single computer. The major computational challenges are in the generation of p-values for MDR through permutation tests but this is not the major focus of our work. Several works have been devoted to improve the efficiency and shorten the computing time in MDR analysis in high-throughput data. We will defer interested readers to the corresponding citations for computing issues in high-throughput MDR analysis. These computational limitations make our strategy appropriate in large scale candidate gene studies, but may be limited in application to genome-wide association studies until further improvements in computing speed are realized or very large-scale computing resources are available.

MDR permutation computing time is largely dependent on the dimension of data sets. In other words, the computing time increases as the number of SNPs and/or the number of subjects increases. Interestingly, the dimension of data does little impact on the computing time of global tests. The computing time for global tests of 1000 p-values is very close to tests of 10 p-values. Several filtration approaches have been proposed and some (ReliefF, SURF and TuRF etc.) have been implemented in the MDR software (http://www.epistasis.org). In this work, we utilize ReliefF for filtration. There have been other filtration techniques proposed in literature. For instance, [34] introduced entropy-based information gain to search and evaluate interactions among risk factors. The current MDR software [8] provides ReliefF, entropy, chi-square test, etc. about 10 filtration methods. The global tests could be integrated in the workflow with other filtration techniques, although the comparison and evaluation of all filtration technique requires more research attention.

## Appendix 1

Simulation of correlated p-values

We generate correlated Beta variables using the method proposed by [30]. According to Bayesian theory, random variables from a Beta prior and a Beta-Binomial conjugate function will yield correlated random deviates whose marginal distribution is also Beta. Firstly, randomly generate a variable *K* from K~Beta−Binomial(v,α,β) where *α* and *β* are the shape parameters. Conditioning on K=k, generate *P* deviates from Beta(α+k,v+β−1). By integrating on *K*, the *P* deviates have unconditional marginal distribution as Beta(α,β) and the correlation coefficient among *P* deviates is ρ=v/(v+α+β). In this paper, we simulated different correlation coefficient with constant ρ=0.2,08 or as a random variable from ρ~Beta(2,5) and ρ~Uniform(0.1,0.9) respectively.

In the above method, ρ=v/(v+α+β) can be written as v=(α+β)ρ/(1−ρ) but algorithm to generate Beta-Binomial with non-integer *v* is not widely available. As a result, we use an alternative method to generate correlated uniform distributions . In essence, correlated uniform variables, *U*, with a random correlation matrix Σ can be generated by transforming multivariate normal variables *X* using formula U=F(X) where *F* is the CDF of the standard normal distribution. First, we generated a positive definite covariance matrix, Σ_0_ with randomly selected eigenvalues and randomly generated orthogonal matrix as eigenvectors(R clusterGeneration package). Let σ_ij_ be the component in Σ_0_. We can convert the covariance matrix, Σ_0_ to correlation matrix Σ with components $r_{\mathrm{ij}}=\frac{\sigma_{\mathrm{ij}}}{\sqrt{\sigma_{\mathrm{ii}}\sigma_{\mathrm{jj}}}}$. To ensure the correlation is invariant to transformation, we need to adjust correlation matrix Σ into $\sum_{}^{\mathrm{adj}}=2\sin(\pi \sum /6)$. Perform Choleski factorization to generate $C={\left(\sum^{\mathrm{adj}}\right)}^{\frac{1}{2}}$. Generate a vector of i.i.d. standard normal variables, *X*_0_. Let $X=X_{0}*C$ and U=F(X). As a result, the variables *U* are correlated uniform variables with correlation matrix Σ.

## Competing interests

There are no competing interests to this work.

## Authors’ contributions

HD and MB conceived of the study. AMR aided in study design and MDR method. HD and AMR performed the simulations and data analysis. MB, RG, and SL performed the clinical data collection, genotyping and interpretation of case study findings. All authors contributed to the manuscript writing. All authors read and approved the final manuscript.
